# Primary somatosensory contribution to action observation brain activity—combining fMRI and cTBS

**DOI:** 10.1093/scan/nsw029

**Published:** 2016-03-15

**Authors:** Nikola Valchev, Valeria Gazzola, Alessio Avenanti, Christian Keysers

**Affiliations:** ^1^Department of Neuroscience, University of Groningen, University Medical Center Groningen, Antonius Deusinglaan 2, 9713 AW Groningen, The Netherlands; ^2^The Netherlands Institute for Neuroscience, Royal Netherlands Academy of Arts and Sciences (KNAW), Meibergdreef 47, 1105 BA Amsterdam, The Netherlands; ^3^Department of Psychology, University of Amsterdam, Weesperplein 4, 1018 XA Amsterdam, The Netherlands; ^4^Department of Psychology and Centro studi e ricerche in Neuroscienze Cognitive, University of Bologna, Cesena Campus, Cesena 47521, Italy,; ^5^Istituto di Ricerca e Cura a Carattere Scientifico Fondazione Santa Lucia, Rome 00179, Italy

**Keywords:** SI, BA1/2, TMS, action observation, mirror neuron system

## Abstract

Traditionally the mirror neuron system (MNS) only includes premotor and posterior parietal cortices. However, somatosensory cortices, BA1/2 in particular, are also activated during action execution and observation. Here, we examine whether BA1/2 and the parietofrontal MNS integrate information by using functional magnetic resonance imaging (fMRI)-guided continuous theta-burst stimulation (cTBS) to perturb BA1/2. Measuring brain activity using fMRI while participants are under the influence of cTBS shows local cTBS effects in BA1/2 varied, with some participants showing decreases and others increases in the BOLD response to viewing actions *vs* control stimuli. We show how measuring cTBS effects using fMRI can harness this variance using a whole-brain regression. This analysis identifies brain regions exchanging action-specific information with BA1/2 by mapping voxels away from the coil with cTBS-induced, action-observation-specific BOLD contrast changes that mirror those under the coil. This reveals BA1/2 exchanges action-specific information with premotor, posterior parietal and temporal nodes of the MNS during action observation. Although anatomical connections between BA1/2 and these regions are well known, this is the first demonstration that these connections carry action-specific signals during observation and hence, that BA1/2 plays a causal role in the human MNS.

## Introduction

Functional magnetic resonance imaging (fMRI) evidences a network of regions, agnostically dubbed ‘shared circuits’, activated both during action perception (observation or listening) and execution. [e.g. Grezes *et al.*, 2003; Gazzola *et al.*, 2006, 2007; Dinstein *et al.*, 2007; Filimon *et al.*, 2007; Gazzola and Keysers, 2009; Ricciardi *et al.*, 2009; Turella *et al.*, 2009; see Caspers *et al.* (2010) and Molenberghs *et al.* (2012) for meta-analyses]. Shared circuits include, in addition to occipital and temporal regions associated with vision and audition, two additional groups of areas. One, associated with the motor system, includes dorsal and ventral premotor cortices and the inferior parietal lobe. Because mirror neurons were recorded in these regions (Gallese *et al.*, 1996; Umiltá *et al.*, 2001; Kohler *et al.*, 2002; Keysers *et al.*, 2003; Cisek and Kalaska, 2004; Fogassi *et al.*, 2005; Fujii *et al.*, 2008; Rozzi *et al.*, 2008; Mukamel *et al.*, 2010), this group has been called the (putative) mirror neuron system (pMNS). The other group, mainly associated with the somatosensory system, includes posterior regions of the primary (Brodmann Area 1 and 2 in particular, BA1/2) and secondary somatosensory cortex (SII). Somatosensory cortices may therefore contribute to perceiving others in general (Adolphs *et al.*, 2000; Bufalari *et al.*, 2007; Valeriani *et al.*, 2008; Keysers *et al.*, 2010; Bolognini *et al.*, 2011; Bolognini *et al.*, 2013; Bolognini *et al.*, 2014), and their actions in particular (Avenanti *et al.*, 2007; Gazzola and Keysers, 2009; Caspers *et al.*, 2010; Keysers *et al.*, 2010; Jacquet and Avenanti, 2015). As neuroscience embraces that cognition results from the interplay of multiple regions, the challenge becomes to understand the interplay between the components of the shared circuits. BA1/2 has strong direct anatomical connections with posterior parietal regions of the pMNS and strong indirect connections with premotor regions of the pMNS (Keysers *et al.*, 2010). The connections between BA1/2 and dorsal premotor cortex are mainly mediated via posterior parietal regions PE and PFG (Rizzolatti *et al.*, 1998; Geyer *et al.*, 2000). Those between BA1/2 and the ventral premotor cortex are mainly mediated through SII, PF and the intraparietal cortex (AIP and VIP), although some direct connections also exist (Rizzolatti *et al.*, 1998; Geyer *et al.*, 2000; Gerbella *et al.*, 2011). The critical question at hand, to help understand the neural basis of action observation, is therefore whether BA1/2’s anatomical connections with the pMNS convey information about observed actions.

To address this question we use continuous theta-burst stimulation (cTBS), a repetitive transcranial magnetic stimulation (TMS), to perturb BA1/2 activity, and measure through fMRI whether this has remote effects on action-observation-specific activity in the pMNS (Driver *et al.*, 2009; Siebner *et al.*, 2009; Reithler *et al.*, 2011). Previous TMS/fMRI studies showed the power of this combination of TMS and fMRI: the intensity of TMS stimulation applied on the frontal eye-field was found to correlate with changes of fMRI activity measured in the early visual cortex. These remote effects have been interpreted as strong evidence for a causal backward influence from the frontal eye-fields to the visual cortex (Ruff *et al.*, 2006; Hubl *et al.*, 2008; Driver *et al.*, 2009; Reithler *et al.*, 2011). By analogy, if we were to find changes in activity in the pMNS that correlated with cTBS-induced activity changes in BA1/2, this would provide similarly strong evidence for a causal contribution of BA1/2 to information processing in the pMNS. The long lasting effects of cTBS on brain activity (∼15–50 min post-stimulation; Huang *et al.*, 2005 , 2011; Ishikawa *et al.*, 2007; Wischnewski and Schutter, 2015) made it is possible to perturb BA1/2 before the fMRI measurement, and yet measure brain activity while still under cTBS perturbation. This avoids the technical (e.g. interrupting scanning to deliver magnetic pulses, using larger head coils to accommodate the TMS) and theoretical (confounding the effect of cTBS over BA1/2 with the sensory experience of TMS stimulation) problems of combining TMS and fMRI online.

Although in behavioral and neurophysiological experiments cTBS is generally assumed to have a net ‘suppression’ effect on the neural activity under the coil, the effect of cTBS is actually a complex combination of suppression and excitation and is highly variable across individuals (Gentner *et al.*, 2008; Iezzi *et al.*, 2008; Ridding and Ziemann, 2010; Huang *et al.*, 2011; Hamada *et al.*, 2012; Gratton *et al.*, 2013; Hartwigsen *et al.*, 2013; Vidal-Piñeiro *et al.*, 2014; Wu *et al.*, 2014), in particular when the effect of cTBS (and of other ‘inhibitory’ TMS protocols) (Arfeller *et al.*, 2012) is assessed locally through fMRI signal. Accordingly, we do not expect cTBS to cause a change in local activity in BA1/2 that is homogeneous across individuals (i.e. all participants showing inhibition or all showing excitation). Instead, the fMRI signal in BA1/2 should show an increase in variance across individuals due to some subjects showing inhibition and other excitations. Accordingly, we measure for each participant how cTBS (compared with SHAM) changes brain activity in BA1/2, and then examine whether voxels in the pMNS show changes in brain activity in the same direction. To explore whether information relative to action observation is transmitted from BA1/2 to the pMNS, we examined this relationship for a contrast between activity triggered by seeing a goal-directed action and that triggered by a non-action stimulus.

## Materials and Methods

### Participants

Twenty-four participants took part in the study, but six failed to complete all three sessions (two because of excessive resting motor threshold (rMT)  >64%, two because of voluntary drop-out and two because of light headaches on Day 2—a SHAM session for both), and one was excluded because his stimulation point fell posterior to BA1/2. The final 17 subjects (six female, 20.9 y  ±  1.95 s.d.) were right handed (Edinburgh handedness inventory mean score: 82.2 y  ±  17.6 s.d.) (Oldfield, 1971), had normal or corrected-to-normal visual acuity; had no neurological, psychiatric or other medical problem, nor contraindications to cTBS (Rossi *et al.*, 2009, 2011) or fMRI and were naïve to the purposes of the experiment. Full debriefing was provided at the end of the third session. Participants gave written informed consent and received monetary compensation (8€/h). Procedures were approved by the Medical Ethical Committee of the University Medical Center Groningen.

### Resting motor threshold

rMT was determined by stimulating the left motor cortex and recording motor-evoked potentials (MEPs) from the right first dorsal interosseus (FDI) by means of a TMSi-Refa 16-channels amplifier (TMS international, Oldenzaal, The Netherlands). Electromyographic signals were sampled at 5 kHz and band-pass filtered (20–1000 Hz). Pairs of silver/silver-chloride electrodes were placed over the FDI in a belly-tendon montage. The TMS scalp position was chosen to produce maximum MEPs amplitude in the FDI muscle. The rMT was defined as the weakest stimulation inducing MEPs  ≥ 50 µV with 50% probability (Rossini *et al.*, 1994, 2015).

### cTBS protocol

Bursts of three TMS pulses were delivered at 50 Hz, each burst repeated every 200 ms (5 Hz) for a total of 600 pulses in 40 s (Huang *et al.*, 2005; Franca *et al.*, 2006; Bertini *et al.*, 2010). Stimulation was administered with a 70-mm figure-of-eight coil connected to a Magstim Rapid2 (Magstim, Wales, UK). Sham stimulation was delivered with the same parameters but through a sham coil (Magstim), which produces sounds and sensations on the skin that approximate those of the active coil. Pulse intensity was set at 80% of rMT (=47.35%  ±  5.06 s.d. of maximum stimulator output).

### MRI data acquisition

Images were acquired with a Philips Intera 3T Quaser with a 8Ch synergy SENSE head coil. Functional images: 28 AC–PC aligned axial gradient-echo slices, 4.5 mm thickness, no gap, 3.5 x 3.5 mm in plane, interleaved slice acquisition, single shot EPI; TE = 28 ms, TR = 1.33 s. T1 weighted structural scans: TR = 7.657 ms, TE = 3.54 ms, flip angle =  8 deg, 1×1×1 mm voxel size.

### Observation task

**Fig. 1.**
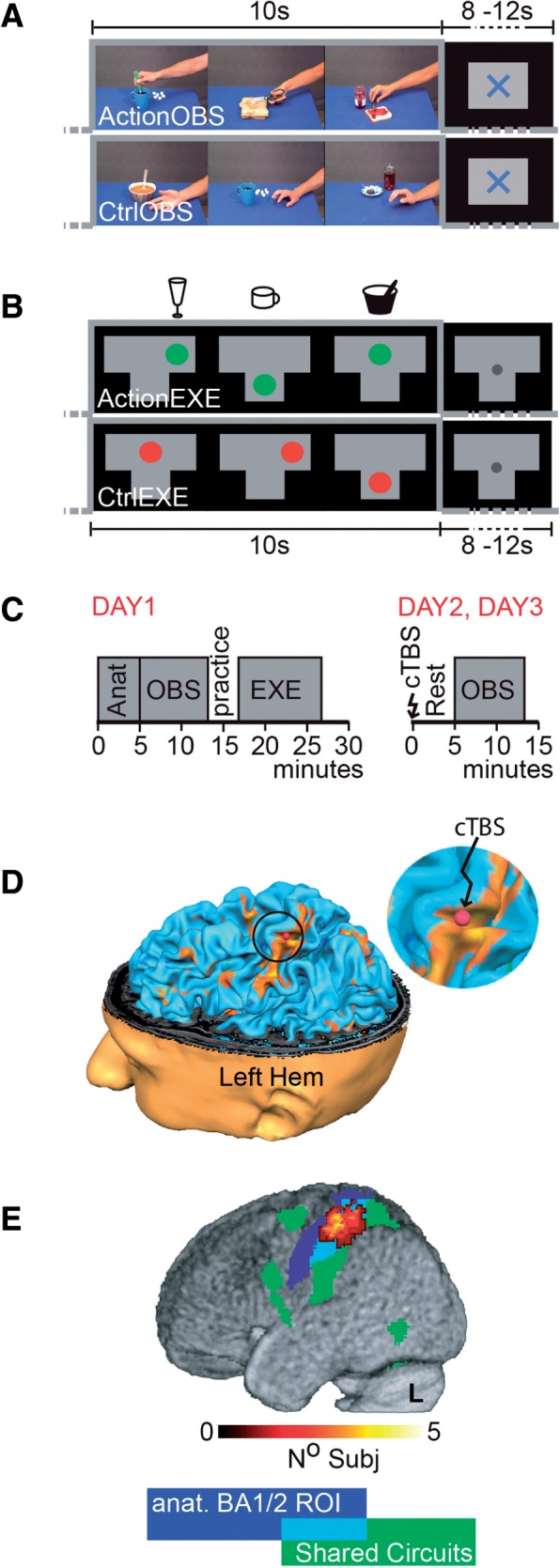
Experimental stimuli and design. (**A**) Observation task—timing with example frames of an ActionOBS and CtrlOBS block. All ActionOBS stimuli started with the actor’s right hand entering from the right side of the screen, moving toward an object already present on the table and acting on it. In the CtrlObs videos, the hand moved close to the same objects but did not interact with it. A 8–12 (random) s fixation cross separated blocks of different conditions. (**B**) Execution task*—*A spoon in a bowl, a wine glass and a coffee cup (sketches above the timelines) were positioned on three locations of a T-shaped table placed over the participants’ abdomen. During each 10 s block subjects were required to use the spoon as to scoop soup from the bowl, to swirl the wine glass or to grasp the coffee cup, each with their right hand and in randomized order. Instructions were back-projected on a screen (shown in the timeline): a green dot appeared on a drawing of the table, in the location corresponding to the object subjects had to act upon. The circle shrank three times to indicate the action duration (matched to the duration of the ActionObs stimuli). In the CtrlExe subjects had to track the same (although red instead of green) dot movements with their gaze, but without interacting with the objects. (**C**) Timeline of the three experimental days. (**D**) Location of the stimulation site (red dot) for one subject as seen on the neuronavigation system. (**E**) Overlap of the ROIs across subjects, superimposed to the shared circuit map (see also Figure 2A) in green and the anatomically defined left BA1/2 in blue. Shade of warm color shows how many subjects’ ROI included this voxel.

Thirty-six distinct hand action interactions (ActionObs) and thirty-six matching control movements (CtrlObs) (Supplementary Table S1 and Arnstein *et al.*, 2011) were recorded using a digital video camera (Sony DSRPDX10P), prepared in Adobe Premiere (www.adobe.com) and presented using Presentation (Neurobehavioral Systems, Davis, CA) (Figure 1A). Three movies of the same category formed a 10-s block and 12 blocks of each condition were presented in a semi-randomized fashion (i.e. no more than two repetitions of the same condition in a row). At the end of the approximately 8 min session, subjects answered four questions that tested whether subjects watched the movies carefully or not.

### Execution task

Similarly to Arnstein *et al.* (2011) (and Gazzola *et al.*, 2007; Gazzola and Keysers, 2009), subjects were requested via back-projected instruction to either act upon objects presented on a T-shaped table on their abdomen (ActionExe), or track a moving dot on the screen (CtrlExe) with their gaze (Figure 1 legend for more details) (Figure 1B). Each condition was repeated 12 times in a semi-randomized order with a random inter-trial interval of 8–12 s. Subjects practiced and rehearsed the task before the beginning of this approximately 8 min fMRI session.

### Experimental protocol

The experiment was distributed over 3 days (Figure 1C).

***Day1. Localization of shared circuits and BA1/2***. On the first experimental day, subjects performed the observation and execution tasks while in the MRI. To prepare for neuronavigation (Brain Innovation, Maastricht, The Netherlands), the T1 anatomical scan was acquired prior to functional tasks and immediately processed. After scanning, we evaluated the rMT and saved (using neuronavigation) the corresponding optimal scalp position for further use. Subjects showing high rMT were invited to end the experiment (*n* = 2, rMT > 64%).

***Day2 and Day3. Sham and cTBS***. Days 2 and 3 were equal in everything but the type of cTBS protocol randomly assigned to participants: nine received sham during Day 2 and cTBS on Day 3. The opposite was true for the remaining eight. Comparing results in participants experiencing cTBS first, and those experiencing SHAM first, revealed no significant differences at *P*_unc_ < _ _0.001. Each day started with the re-assessment of the rMT on the scalp position saved during Day 1 and the localization of our BA1/2 target point (see the ‘Target site selection and neuronavigation’ section). Subjects were then taken to the MRI preparation room, seated in the MRI bed (previously moved to the preparation room), asked to relax trying not to move their right (contralateral to stimulation) arm while stimulated with the cTBS (or SHAM) protocol. The experimenter then helped subjects to lay down to minimize subjects’ movements after cTBS (Gentner *et al.*, 2008; Iezzi *et al.*, 2008; Todd *et al.*, 2009), and pushed the bed into the scanner. Within 6 min (5.2 min ± 0.41 s.d.) the fMRI scanning sequence was initiated, to capture the cTBS influence on action observation when cTBS after effects are thought to reach their maximum level (Huang *et al.*, 2005).

***Target site selection and neuronavigation***. Subjects’ head was reconstructed in 3D for neuronavigation using BrainVoyager (BV, Brain Innovation) from their T1 images. Functional data from Day 1 were pre-processed in BV (3D motion correction, FWHM 6-mm filter spatial smoothing, temporal filtering), and resulting images were co-registered to the T1. Unnormalized data were used to identify our target point (Figure 1D): the section of the somatosensory cortex that (a) belonged to the cluster resulting from inclusively masking the contrast ActionOBS–CtrlObs (visualized for most of the subjects at *P*_unc _ < _ _0.001, but threshold was lowered in some cases) with the binary map from ActionExe–CtrlExe (all subjects at *p*_unc__ _< 0.001, minimum cluster size 10, *q*_FDR__ _<_ _ 0.05), and (b) fell within the anterior bank of the post-central sulcus and the adjacent crown of the postcentral gyrus (Geyer *et al.*, 1999; Grefkes *et al.*, 2001). Through neuronavigation, the target point was marked on an EEG cap worn by the participant. Mean Talairach coordinates (±s.d.) for the activation target site were: −43 ± 5.52, −31 ± 5.98, 54 ± 5.49 (transformed in MNI using http://imaging.mrc-cbu.cam.ac.uk/downloads/MNI2tal/: −43 −35 57).

***Data pre-processing and analyses***. Except for neuronavigation, all analyses were carried out with SPM8 (Wellcome Department of Imaging Neuroscience, London, UK). Slice time-corrected EPI volumes were aligned to the mean EPI image from all 3 days. The T1 gray matter segment was co-registered to the mean EPI, and used to determine normalization parameters applied to all EPI (2× 2× 2 mm) and structural (1×1×1 mm) images. EPIs were then smoothed with an 8-mm FWHM Gaussian kernel.

At the first (subject) level, for each day separately, ActionObs and CtrlObs were modeled with separate predictors as boxcar functions convolved with the hemodynamic response function. The same was done for ActionExe and CtrlExe. Six movement parameters, which never exceed the original voxel size, were included as predictors of no interest. Second level analyses were performed as described in the results. We used this two level approach to examine whether cTBS changed the reliability of brain activity (thereby increasing the residual error at the first level, see Supplementary Materials) or shifted brain activity up or down throughout the cTBS session (thereby increasing variance across participants at the second level, see main text). Brain maps were thresholded at *P_uncorrected_* < *_ _*0.001, and *P*_FDR__ _< _ _0.05 (whichever most stringent; see Supplementary Section ‘Statistical maps thresholding considerations’).

***BA1/2 ROI definition***. For each subject individually, an 1 cm diameter sphere, centered on the MNI transformed target point, was built with Marsbar (Brett *et al.*, 2002), then intersected with both the BA1/2 anatomical ROI (Eickhoff *et al.*, 2005 , 2006, 2007), and the individual gray matter (Figure 1E).

**Fig. 2.**
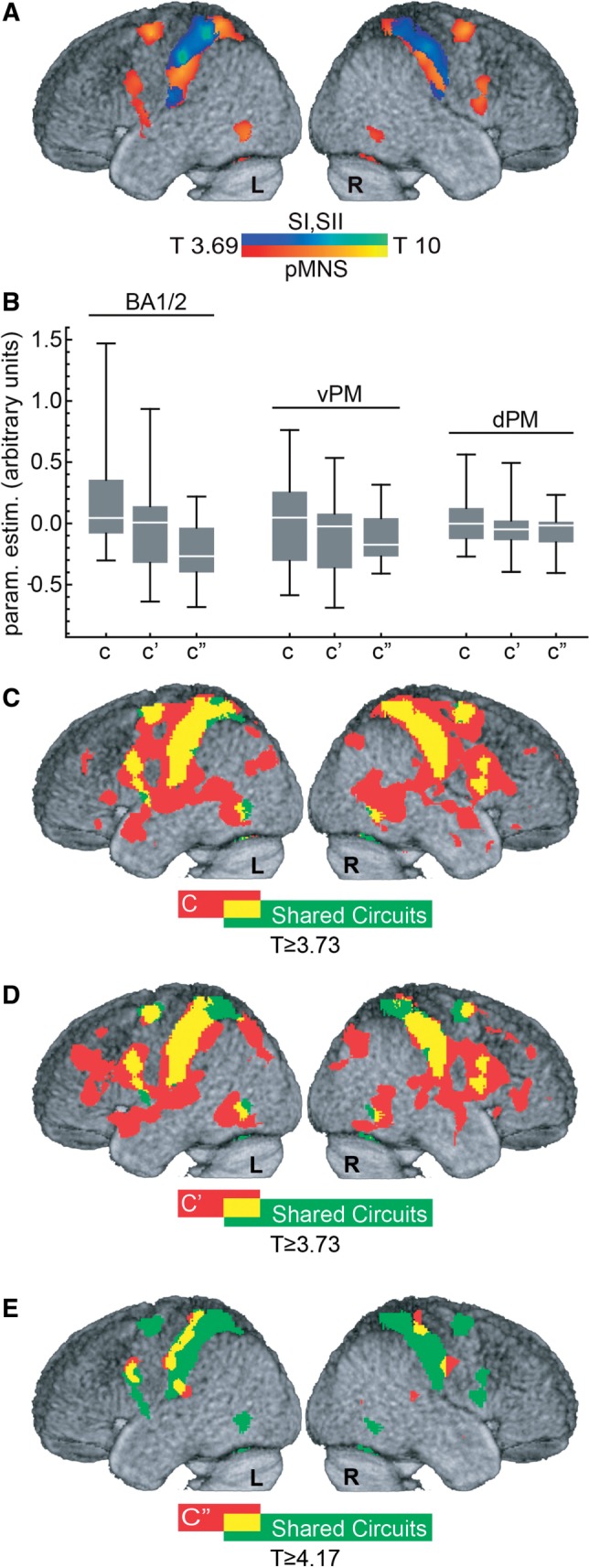
Shared circuits, target site and remote effect of cTBS on SI. (**A**) Localization of the shared circuits (*P* ≤ 0.001, *T*_(16)_ ≥ 3.69, results survive *q*_FDR  _≤ _ _0.05). Warm colors show areas classically associated with the pMNS, cold colors, somatosensory regions. (**B**) Box and whisker plot illustrating the spread of C, C′and C″ values as a function of brain region (whiskers: min and max, box: Q1 and Q3, midline: median). (**C**–**E**) Regression analysis results for C, C′ (both *P* ≤ 0.001, *T*_(16)_ ≥ 3.69, also survives *q*_FDR_ ≤ _ _0.05) and C″ (*q*_FDR _ ≤ _ _0.05, *T*_(16)_ ≥ 4.17), respectively. In green the shared circuits as defined in A. In red voxels with significant C, C′ or C″ regression values. In yellow the overlap between the shared circuits and the regression results.

***Premotor ROIs definition*.** Left BA6 and BA44 (Anatomy toolbox for SPM; Eickhoff *et al.*, 2005 , 2006, 2007) were at first combined in a single BA6/44 ROI. Based on visual inspection of our group averaged anatomy, the study of Tomassini *et al.* (2007), and on the Harvard–Oxford cortical atlas (http://www.cma.mgh.harvard.edu/fsl_atlas.html), the BA6/44 ROI was then split in three: voxels with −13  ≤  × ≤ +13 (in MNI) were combined into the supplementary motor, voxels not belonging to SMA with *z*  ≥  48, combined into dorsal premotor and those with *z* < 48 into ventral premotor ROI. Intersecting these anatomical ROIs with the results of the shared circuit localizer generated dPM and vPM ROIs used to quantify the effect of cTBS over BA1/2 on the core premotor nodes of the pMNS (Figure 2B and Supplementary Figure S4).

## Results

### Shared circuits

Shared circuits were localized at group level from data acquired on Day 1: the contrast ActionObs–CtrlObs (*T*_(16)_ ≥ 3.69, *P*_unc__ _≤ _ _0.001, minimum cluster size 10 and *q*_FDR__ _< _ _0.05) was explicitly masked with results from ActionExe–CtrlExe thresholded at *T*_(16)_ > 3.69 (*P*_unc__ _< _ _0.001, minimum cluster size 10 and *q*_FDR__ _< _ _0.05). As expected, shared circuits included (Table 1) regions of the pMNS (premotor and posterior parietal cortices; Figure 2A, warm colors) and somatosensory cortices (BA1/2 and SII; Figure 2A, cold colors and Supplementary Figure S1).

**Table 1.** Group shared circuits: ActionObs–CtrlObs and ActionExe–CtrlExe (both at *P* ≤ 0.001, *T*_(16)_  ≥  3.69, *q*_FDR_ _ _≤ _ _0.05).

Cluster size in number of voxels	*T*	*x*	*y*	*z*	Hem	Anatomical description	Cytoarchitectonic description
2886	15.18	−50	−26	44	L	Inferior parietal lobule	Area 2
	13.49	−30	−42	58	L	Postcentral gyrus	Area 2
	12.96	−36	−46	60	L	Superior parietal lobule	SPL
	10.85	−58	−26	34	L	Supramarginal gyrus	IPC
2198	10.22	52	−28	48	R	Postcentral gyrus	IPC
	9.42	42	−32	40	R	Supramarginal gyrus	IPC
	8.12	28	−48	58	R	Superior parietal lobule	Area 2
1500	12.65	−26	−6	58	L	Superior frontal gyrus	Area 6
	9.04	−6	0	48	L	SMA	Area 6
	6.66	−2	8	30	L	Anterior cingulate cortex	
646	15.30	−38	−6	8	L	Insula lobe	
	6.07	−52	4	28	L	Precentral gyrus	Area 44
457	10.59	28	−2	62	R	Superior frontal gyrus	Area 6
333	7.00	54	8	24	R	Inferior frontal gyrus (pars opercularis)	Area 44
	6.40	40	−4	12	R	Rolandic operculum	OP 3
	5.83	40	−4	−2	R	Insula lobe	
217	7.20	32	−58	−26	R	Cerebellum (VI)	
159	4.87	−28	−62	−20	L	Cerebellum (VI)	
99	8.75	−50	−70	−8	L	Inferior occipital gyrus	
82	5.03	−12	−20	−2	L	Thalamus	
74	4.54	−24	10	−2	L	Putamen	
69	5.90	−6	−78	−4	L	Linual gyrus	Area 17
49	5.36	54	−62	−10	R	Inferior temporal gyrus	hOC5

From left to right: the cluster size in number of voxels; the *T* values, the MNI coordinates in mm, the hemisphere, the anatomical description and, when available, the cytoarchitectonic description (as given by the Anatomy toolbox) of the local maxima within the cluster.

### Effect of cTBS on BA1/2

To examine cTBS effects on action observation processing (operationalized using ActionObs–CtrlObs), in the target location, we extracted from each participant (i), the parameter estimates of the contrast C*_i_* **= * *cTBS_(ActionObs__–__CtrlObs)_−SHAM_(ActionObs__–__CtrlObs)_ from the participant’s BA1/2 targeted ROI (Figure 2B). As expected, the group overall showed no clear main cTBS effect (*P* > 0.09, *t*-test H_0_: C = 0), and some participants showed a reduction of the signal in the somatosensory cortex (C < 0; parameter estimates in arbitrary units from −0.3 to −0.04; *n* = 8), others an increase (*C* > 0; parameter estimates in arbitrary units from +0.05 to +1.47; *n* = 9). To examine whether this variability was due, at least in part, to the effect of cTBS, or only to random fluctuations between two scanning sessions, we calculated a similar contrast between the cTBS and LOCALIZER day, C*_i_*′ =  cTBS_(ActionObs__–__CtrlObs)_−LOCALIZER_(ActionObs__–__CtrlObs)_ and the SHAM and LOCALIZER day, C*_i_*″ = SHAM_(ActionObs__–__CtrlObs)_−LOCALIZER_(ActionObs__–__CtrlObs)_. If cTBS has an effect on BA1/2 that varies from excitatory to inhibitory depending on subjects (Gangitano *et al.*, 2002; Hamidi *et al.*, 2009; Ridding and Ziemann, 2010; Hamada *et al.*, 2012), C′ should have a wider distribution than C″—with participants showing inhibition widening the distribution to the left and those showing excitation to the right tail of the distribution of C′ compared with C″.

As expected, the standard deviation of contrasts involving cTBS (*σ*(C) = 0.43, *σ*(C′) = 0.39) was larger than those of the contrast not involving cTBS (*σ*(C″) = 0.26), with cTBS leading to an increase of standard deviation of over 50% (*σ*(C)/*σ*(C″) = 1.67, *σ*(C′)/*σ*(C″) = 1.52). A permutation test, in which we randomly permute (within each subject) the labels for C, C′ and C″ 10 000 times revealed that only 90 of these 10 000 random permutations exceeded the values observed in our real data (both *σ*(C)/*σ*(C″) > 1.67 and *σ*(C′)/*σ*(C″) > 1.52). This confirms that cTBS increased between-subject variance in our contrast values (*P* < 0.009). An alternative approach to testing whether the contrasts involving cTBS (C, C′) differ in distribution from those not involving cTBS (C″) is to use the non-parametric paired-sample Kolmogorov–Smirnov test of equality of distribution. The test confirmed that C and C′ came from similar distributions (*P*_(one tailed)_ > 0.19), but both come from distributions that differ from those of C″ (C *vs* C″, *P*_(one tailed)_ < 0.0015; C′ *vs* C″, *P*_(one tailed)_ < 0.04).

### The remote effects of cTBS

We used a regression analysis to explore whether voxels of the pMNS receive information relating to action observation from BA1/2, and whether this information is sensitive to cTBS. Importantly, we use the contrast C to isolate brain activity that relates to action observation. If a given voxel (*j*) receives action-specific excitatory input from BA1/2, remote effects should mirror local effects, with participants for whom cTBS increased activity in BA1/2 (contrast C_BA1/2__ _> _ _0) showing increased activity in this voxel *j* (C*_j_*_ _> _ _0), and participants for whom cTBS reduced activity in BA1/2 (C_BA1/2__ _< _ _0) showing reduced activity in voxel *j* (C*_j_*_ _< _ _0). Hence, we computed a general linear model (GLM) of the form C*_j_*_,_*_i_*_ _= _ _*a_j_* * C_BA1/2,_*_i_*_ __ _+ _ _error*_i_*, and tested H_0_: *a_j_*_ _ ≤ _ _0 against the alternative hypothesis H_1_: *a_j_*_ _> _ _0. This regression analysis revealed a large bilateral network encompassing the dorsal and ventral premotor cortex, and the rostral inferior parietal lobule of the pMNS, BA1/2, primary motor and regions of the middle temporal gyrus (MTG) (Table 2 and Figure 2C). Among the 8792 voxels localized to belong to shared circuits on Day 1 (Figure 2A, and green in 2C–E), 6373 (72.5%, Figure 2C yellow) were found to have activation changes (C*_j_*) significantly predicted by changes associated with cTBS on BA1/2 (C_BA1/2_) (see also Supplementary Figure S2). Importantly, although the remote effects predicted by C_BA1/2_ were not restricted (red in Figure 2C) to shared circuits, there was a notable topographic similarity between the spatial maps retrieved by this regression analysis and shared circuits (spatial correlation of the *t*-maps, *r* = 0.38, *P* < 10 ^−6^). To test for inhibitory connections between BA1/2 and target voxels, we tested for the presence of negative relationships (H_1_: *a* < 0), but found no significant results (*q*_FDR__ _> _ _0.05). If BA1/2 sends inhibitory connections to voxel *j*, and if this inhibition is metabolically more costly than the activity it inhibits, inhibitory connections may have been included in the significant positive contrast (H_0_: *a*  ≤  0) (Attwell and Iadecola, 2002).

**Table 2.** Remote effect of cTBS

Cluster size in number of voxels	*T*	*x*	*y*	*z*	Hem	Anatomical description	Cytoarchitectonic description
Contrast C (*P* ≤ 0.001, *T*_(15)_ ≥ 3.73, *q*FDR ≤ 0.05**):** cTBS_(ActionObs__–__CtrlObs)_−SHAM_(ActionObs__−__CtrlObs)_							
22 792	17.45	−54	−24	50	L	Inferior parietal lobule	Area 1
	15.70	−46	−30	56	L	Postcentral gyrus	Area 1
	11.73	66	−18	24	R	Supramarginal gyrus	IPC
	10.98	40	−34	54	R	Postcentral gyrus	Area 2
	10.69	−60	−20	38	L	Supramarginal gyrus	Area 2
	9.70	−32	−12	60	L	Precentral gyrus	Area 6
	9.45	26	2	66	R	Superior frontal gyrus	Area 6
	9.29	38	−46	62	R	Superior parietal lobule	SPL
5457	10.05	−4	−56	−6	L	Cerebellum (IV–V)	
	9.56	24	−62	−19	R	Cerebellum (VI)	
	7.45	28	−42	−28	R	Cerebellum (IV–V)	
	7.45	−34	−84	30	L	Middle occipital gyrus	IPC
	7.14	−22	−58	−26	L	Cerebellum (VI)	
	6.84	−24	−42	−20	L	Fusiform gyrus	
	6.72	−18	−50	−10	L	Linual gyrus	
	6.61	6	−62	20	R	Cuneus	SPL
426	5.01	8	−20	−2	R	Thalamus	
121	6.21	−20	10	−16	L	Olfactory cortex	
	5.01	−16	4	−20	L	Parahippocampal gyrus	
120	4.64	−34	−22	−28	L	Fusiform gyrus	Hipp (EC)
102	4.73	−30	36	24	L	Middle frontal gyrus	Area 44
96	5.39	42	14	−30	R	Temporal pole	
	4.65	30	2	−26	R	Amygdala	Amyg
80	4.96	−26	−70	46	L	Superior parietal lobule	SPL
74	4.81	−20	−58	12	L	Calcarine gyrus	Area 18
74	5.72	−42	22	4	L	Inferior frontal gyrus (Pars triangularis)	Area 45/45
39	4.43	10	−20	36	R	Middle cingulate cortex	
26	4.00	26	50	8	R	Superior frontal gyrus	
25	4.02	−6	−18	4	L	Thalamus	
24	4.34	−36	30	−12	L	Inferior frontal gyrus (Pars orbitalis)	
22	4.18	26	−20	−20	R	Parahippocampal gyrus	Hipp
21	4.08	6	64	20	R	Superior medial gyrus	
20	5.45	26	18	2	R	Putamen	
18	4.55	10	56	6	R	Superior medial gyrus	
17	4.49	−10	−52	42	L	Precuneus	
17	4.26	8	58	30	R	Superior medial gyrus	
12	4.07	−10	56	8	L	Superior medial gyrus	
11	4.29	32	−40	−12	R	Fusiform gyrus	Hipp
11	4.21	36	30	−16	R	Inferior frontal gyrus (Pars orbitalis)	
Contrast C′ (*P* ≤ 0.001, *T*_(15)_ ≥ 3.73, *q*FDR ≤ 0.05): cTBS_(ActionObs__–__CtrlObs)_−LOCALIZER_(ActionObs__−__CtrlObs)_							

16 107	11.97	−44	−36	61	L	Postcentral gyrus	Area 2
	9.09	−58	−24	44	L	Inferior parietal lobule	Area 2
	8.93	−60	−22	42	L	Supramarginal gyrus	Area 2
	8.90	−60	−22	36	L	Supramarginal gyrus	IPC
	8.67	60	−14	34	R	Postcentral gyrus	Area 1
	8.18	−52	4	16	L	Precentral gyrus	Area 44
	8.14	−46	6	18	L	Inferior frontal gyrus (Pars opercularis)	Area 44
1650	7.42	−20	−10	60	L		Area 6
	6.93	−30	−2	50	L	Middle frontal gyrus	Area 6
	6.68	−4	−20	44	L	Middle cingulate cortex	Area 6
	6.40	−30	−2	44	L	Precentral gyrus	
	5.11	−4	−4	50	L	SMA	Area 6
	4.85	4	−30	40	R	Middle cingulate cortex	SPL
611	6.79	22	36	28	R	Superior frontal gyrus	
	5.35	24	16	52	R	Middle frontal gyrus	
	4.95	20	10	54	R	Superior frontal gyrus	Area 6
520	5.53	28	−78	46	R	Superior occipital gyrus	SPL
	5.33	34	−76	46	R	Superior occipital gyrus	IPC
	5.17	14	−78	50	R	Superior parietal lobule	SPL
	4.68	46	−72	36	R	Angular gyrus	IPC
	3.92	32	−62	50	R	Superior parietal lobule	hIP3
501	7.18	−28	−72	51	L	Inferior parietal lobule	IPC
	4.94	−32	−68	28	L	Middle occipital gyrus	IPC
	4.84	−24	−66	28	L	Superior occipital gyrus	hIP1
494	7.31	−60	−54	−6	L	Inferior temporal gyrus	
	5.10	−48	−68	−14	L	Inferior occipital gyrus	
	4.26	−42	−76	−14	L	Inferior occipital gyrus	hOC4v
385	6.46	60	−54	−6	R	Inferior temporal gyrus	
	4.95	44	−68	−14	R	Inferior occipital gyrus	hOC4v
	4.92	56	−54	8	R	Middle temporal gyrus	
206	7.01	6	6	28	R	Anterior cingulate cortex	
	4.62	−4	6	28	L	Anterior cingulate cortex	
165	5.84	46	36	0	R	Inferior frontal gyrus (Pars triangularis)	Area 45
163	5.06	−10	14	38	L	Middle cingulate cortex	
	4.66	−28	24	46	L	Middle frontal gyrus	
	4.41	−14	16	46	L	Superior frontal gyrus	Area 6
	4.12	−6	24	48	L	SMA	
	4.10	−8	20	52	L	SMA	Area 6
137	4.69	−12	−64	56	L	Precuneus	SPL
61	4.24	0	34	48	L	Superior medial gyrus	
51	4.57	26	−88	24	R	Superior occipital gyrus	Area 18
	4.12	30	−82	26	R	Middle occipital gyrus	
34	5.12	20	−32	8	R	Hippocampus	Hipp
27	4.53	−10	38	20	L	Anterior cingulate cortex	
27	4.27	10	24	20	R	Anterior cingulate cortex	
20	3.95	−4	8	50	L	SMA	Area 6
	3.86	4	8	50	R	SMA	Area 6
20	4.59	30	52	2	R	Middle frontal gyrus	
16	4.52	40	20	−6	R	Insula lobe	
10	3.95	−10	−54	66	L	Precuneus	SPL
Contrast ″ (*q*FDR ≤ 0.05, *T*_(15)_ ≥ 4.17): SHAM_(ActionObs__–__CtrlObs)_−LOCALIZER_(ActionObs__–__CtrlObs)_							

263	6.32	−54	−26	48	L	Inferior parietal lobule	Area 2
	6.23	−60	−18	34	L	Postcentral gyrus	IPC
	5.68	−32	−32	64	L	Postcentral gyrus	Area 1
	5.47	−44	−30	54	L	Postcentral gyrus	Area 2
173	6.53	42	−32	64	R	Postcentral gyrus	Area 1
158	6.25	26	−72	26	R	Superior occipital gyrus	
136	5.76	18	−70	−4	R	Linual gyrus	hOC3v
	4.40	28	−62	−6	R	Fusiform gyrus	Area 18
126	6.38	−56	8	34	L	Precentral gyrus	Area 6
	4.49	−52	6	24	L	Inferior frontal gyrus (Pars opercularis)	Area 44
82	6.37	−62	−28	16	L	Superior temporal gyrus	OP1
	5.63	−52	−22	24	L	Supramarginal gyrus	OP1
59	5.45	60	−12	34	R	Postcentral gyrus	Area 1
	4.20	62	−14	26	R	Postcentral gyrus	Area 3b
59	5.06	54	−2	−12	R	Superior temporal gyrus	
	4.97	50	4	−22	R	Middle temporal gyrus	
54	5.30	−50	−20	−10	L	Middle temporal gyrus	
35	4.99	52	−30	12	R	Superior temporal gyrus	IPC
26	5.70	−40	−30	41	L	Postcentral gyrus	IPC
14	5.62	−26	−2	−24	L	Amygdala	Amyg
13	5.08	−22	−36	−26	L	Cerebellum (IV–V)	
10	4.49	−12	−46	−10	L	Linual gyrus	
	4.47	−8	−48	−10	L	Cerebellum (IV–V)	

Activations resulting from GLM regression analyses calculated for C, C′ and C″. Conventions as in Table 1.

To verify that the results found in the above regression analysis depend on the cTBS effect on BA1/2, rather than on unspecific fluctuations across days, we repeated the analysis using the contrasts C′ and C″ as defined above, and the models C′*_j,__i_*_ _= _ _*a_j_*′ * C′_BA1/2,_*_i_*_ _+ _ _error′*_i_*; C″*_j,__i_*_ _= _ _*a_j_*″*C″_BA1/2,_*_i_*_ _ + _ _error″*_i_* (Figure 2D and E). Results confirmed that regression analyses including the cTBS data (C or C′, Figure 2C and D) evidence a larger network (29 768 voxels for C, 21 310 voxels for C′) influenced by BA1/2 than that restricted to spontaneous fluctuations (1269 voxels for C″, Figure 2E). A chi-square test confirms that regressions using spontaneous fluctuations alone (C″) evidence less significant voxels than regressions leveraging the effect of cTBS (C′ and C″, both *P*_(one tailed)_ < 0.0001). This is true also if only significant voxels within shared circuits are compared (yellow in Figure 2C–E; C: 6373, C′: 4139, C″: 434; C > C″ *P*_(one tailed)_ < 0.0001, C′ > C″ *P*_(one tailed)_  < 0.0001).

In the regression analyses, the FDR correction imposed a higher *t*-threshold on the results for C″ (*T* **≥  4.17) than for C and C′ (both *T*  ≥  3.73). However, even if imposing the stricter threshold (*T* ≥ 4.17; Supplementary Figure S3) on all regressions, C and C′ continue to reveal significantly larger networks than C″ (in shared circuits, C: 5339, C′: 3238, C″: 434; chi-square, *P*_(one tailed)_ < 0.0001).

**Fig. 3.**
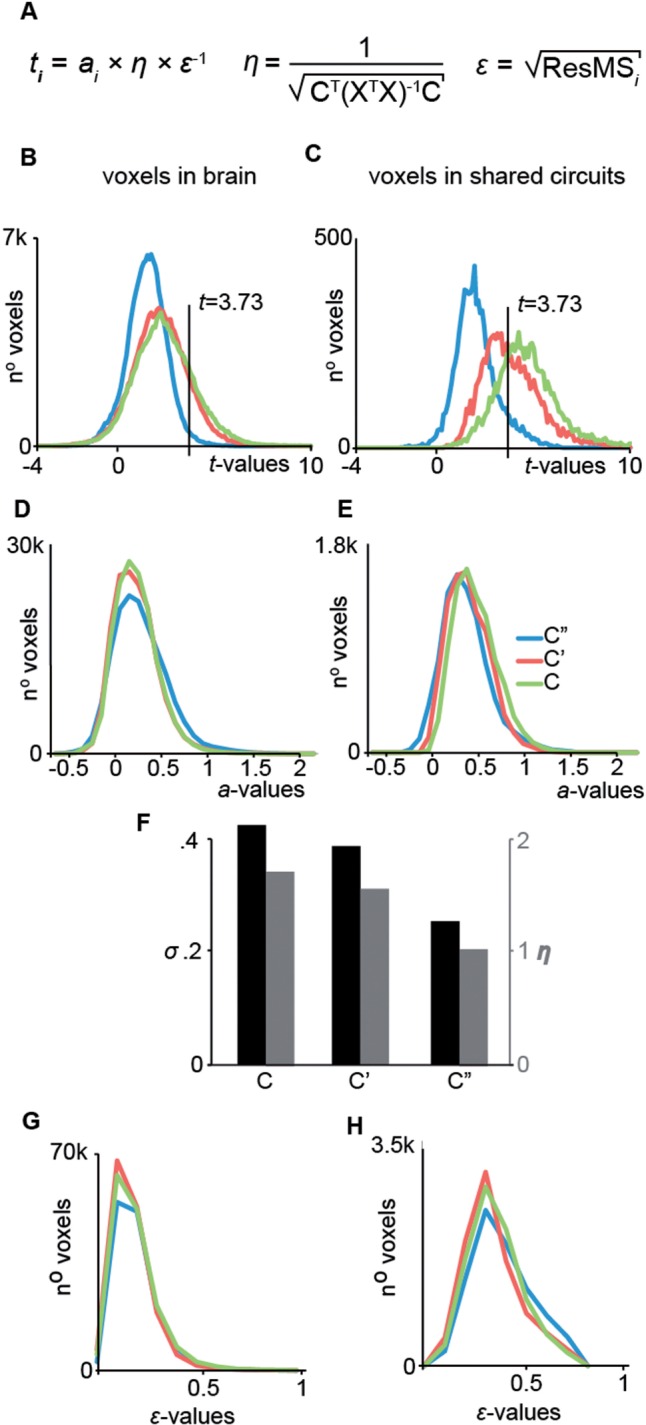
cTBS effect on design efficiency. (**A**) Description of the terms used to calculate *t*-values in a GLM. (**B**, **D**, **F**) Histogram of the *t*-, slope *a*- and error *ϵ*-values over all voxel in the brain. (**C**, **E**, **G**) Same as in B, D and F but over all voxels within the shared circuits. (**H**) Relationship between the efficiency of the design *η*, the standard deviation sigma and the predictor C, C′ and C″.

This analysis has two caveats. First, the chi-square test assumes that voxels are independent, which is untrue due to spatial smoothness of fMRI data. SPM estimates the smoothness of the data, and provides an estimate of the number of resels (independent resolution elements) in the data. If all voxels were independent, there would be as many resels as voxels, instead in our regression based on C, there were 318 voxels per resel. We thus divided all voxel counts in the chi-square 2× 2 contingency table by the SPM estimated 318 voxels/resels, and performed the chi-square tests on resel counts. Results remained significant (all *P* < 0.05). Second, the analysis is dependent on an arguably arbitrary threshold to classify voxels as significant or not in the chi-square table. We therefore plotted the distribution of *t*-values across all voxels for the three regression analyses (Figure 3B and C). This illustrates again, that given a certain threshold (*t* = 3.73, *P*_unc _ <_ _ 0.001) more voxels cross the threshold for C and C′ than for C″. Importantly, the same conclusion would be drawn over a wide range of thresholds, as it is due to an overall shift of the distributions. Non-parametric permutation testing confirms that the distribution for C and C′ have larger medians than that of C″ (*P* < 0.001, 1000 permutations), and this is true over the entire brain (Figure 3B), and within the pMNS (Figure 3C). These results hold even if the permutation test is performed after subsampling one voxel in every 318 to approximately test the differences for resels instead of voxels.

### cTBS induced an increase in design efficiency not in connectivity

The *t*-value in a GLM is the product of three terms (Figure 3A)—the slope *a*, the efficiency *η* and the inverse of the error *ϵ*. Which of the three terms of equation (1) in Figure 3A is responsible for the boost of *t*-value in regressions C and C′ *vs* C″? To test for a change in slope, we first conducted a whole-brain analysis comparing *a'* and *a''* using a multiple regression analysis, which revealed no significant difference (*q*_FDR _ > _ _0.05) on a voxel by voxel basis. Second, we plotted the distribution of the slope as a function of the regression for all voxels or all shared circuit voxels (Figure 3D and E), again showing no evidence for a change in slope. Because the slope of the regression reflects the neural coupling across brain regions (i.e. how much the signal in a target voxel changes when the BOLD signal in SI changes by one unit), the lack of difference in slope suggests that cTBS changed the statistical power of our analyses without changing the neural coupling we try to measure (Supplementary Figure S4, and Supplementary section ‘Changes in connectivity’).

We subsequently examined whether noise (*ϵ*) differed across regressions (i.e. variance across subjects not accounted for by differences in SI), but found no such differences (Figure 3F and G).

As shown above, cTBS increased between subject standard deviation of the contrast that serves as the regressor in the GLMs, with *σ*(C) and *σ*(C′) over 50% higher than *σ*(C″). For a simple regression analysis at the second level, the efficiency *η* of a design increases linearly with the standard deviation of the regressor (via the covariance matrix of the design matrix, X^T^X in Figure 3A, equation (2)). Not surprisingly, the boost of standard deviation in SI triggered by cTBS directly translates into the same boost of 50–70% in the efficiency of the design (Figure 3H). Because of this direct relationship between *σ* and *η*, the permutation test performed on the standard deviations of C, C′ and C″ directly translates to show that the boost in *η* is also significant at *P* < 0.009. Because the efficiency is a property of the regressor, not of the signal in the target voxel, this boost in efficiency is voxel-independent, and therefore directly boosts the *t*-values in all voxels by 50–70%, explaining the shift of *t*-values we identified in Figures 2 and 3.

## Discussion

The experiment aimed to investigate whether during the observation of the actions of others, action specific information processing, as proxied using the ActionObs–CtrlObs contrast, in BA1/2 and pMNS are causally related. We identified the BA1/2 region involved in action observation in each subject, used cTBS to perturb brain activity in this region and then measured the causal effects of this perturbation elsewhere in the brain while subjects viewed the actions of other people.

We expected the local effect of cTBS on BOLD activity in BA1/2 to vary across individuals (Ridding and Ziemann, 2010; Teo *et al.*, 2011; Hamada *et al.*, 2012), as many failed to consistently find a reduction of local activity following ‘inhibitory’ TMS (Chouinard *et al.*, 2003; Lee *et al.*, 2003; Rounis *et al.*, 2005; O’Shea *et al.*, 2007; Hubl *et al.*, 2008; Conchou *et al.*, 2009; Stagg *et al.*, 2009; Havrankova *et al.*, 2010; Ward *et al.*, 2010; Ott *et al.*, 2011; Volman *et al.*, 2011; Arfeller *et al.*, 2012; Noh *et al.*, 2012; van Nuenen *et al.*, 2012). Extracting brain activation from the BA1/2 ROI confirmed our prediction: comparing the cTBS and SHAM sessions revealed that some participants showed a decrease and some an increase in the ActionObs–CtrlObs contrast. Importantly, comparing changes in brain activity induced by cTBS with spontaneous fluctuations across days without cTBS revealed that cTBS had significantly broadened the distribution of action observation-related brain activity across participants. We then leveraged the increase in spread to explore BA1/2 connectivity, by identifying voxels in the brain for which activity changes were predicted by those experimentally induced in BA1/2. This analysis revealed a network of regions encompassing 70% of shared circuit voxels (as identified using our localizer), including areas associated with the pMNS. Importantly, analyses revealed that the cTBS did not alter the actual connectivity between BA1/2 and pMNS regions, but simply increased the efficiency with which such connections can be detected (Figure 3): using cTBS to increase the variance between participants gave the regression analysis more ‘traction’ to detect these connections.

Anatomical connections between BA1/2 and the ipsilateral premotor and inferior parietal nodes of the monkey MNS (Keysers *et al.*, 2010) suggest a human anatomical routes for the causal influence of BA1/2 on the premotor and parietal nodes of the pMNS during action observation. Connections between left and right BA1/2 provide a basis for the strong effects we measured in the right, unstimulated hemisphere. That somatosensory, premotor and parietal brain regions are causally interconnected while participants perform actions is well established (Pearson *et al.*, 1971; Pavlides *et al.*, 1993; Gordon *et al.*, 1995; Aschersleben *et al.*, 2001; Schabrun *et al.*, 2008; Franklin and Wolpert, 2011). The contribution of our study is to propose and test that this interconnection is also relevant during the observation of the actions of others, as this link had so far been neglected: most of the most authoritative reviews on the neural basis of action observation either do not mention the somatosensory system at all (Cattaneo and Rizzolatti, 2009), or see it as an ‘additional’ system merely receiving information from the pMNS (Rizzolatti and Sinigaglia, 2010). This stance implicitly suggests that the anatomical connections from BA1/2 to premotor and posterior parietal regions, that are well documented during action execution, are dormant during action observation. Our data challenge this implicit belief. First, that a manipulation of activation in BA1/2 carries over to premotor and posterior parietal locations suggest that information also flows in the direction from BA1/2 to the classical pMNS system during action observation. Second, by tracking the contrast ActionObs–CtrlObs our analyses show that action-observation-specific information is transferred in the direction from BA1/2 to the pMNS. Importantly, because our CtrlObs condition showed the same hand moving close to the same object as during ActionObs, we disentangle unspecific activation triggered by the sight of hands and objects, from more specific activation triggered when observing a hand acting on the object. Our data therefore invite us to include BA1/2 in the pMNS and suggest that information about the expected somatosensory consequences of the observed action is also part of the simulations computed by the network. The realization that such causal interactions integrate somatosensory and motor representations during action observation nicely fit with contemporary notions of the motor system as being sensorimotor (Gallivan and Culham, 2015) and studies that show that the somatosensory consequences of observed actions influence activation in the action observation network (Morrison *et al.*, 2013).

Given that we employ a regression analysis across days, is it possible that we actually identified a correlation between spontaneous fluctuations across time in both regions? That cTBS changed the variance between participants’ brain activity in the BA1/2 and thereby enabled the regression analyses to evidence a network of meaningful connections that was much wider, jointly advocates that the results of our study reflect, at least in part, a causal influence from cTBS on BA1/2 to the pMNS.

This study has three limitations. First, it lacks of a control area and further studies are thus needed to understand the specificity of the BA1/2 perturbation effects. Second, it cannot establish whether the cTBS-triggered activation changes are functionally relevant for action perception (Avenanti *et al.*, 2013b; Urgesi *et al.*, 2014). To address this question, in a separate experiment, participants saw a box being lifted and had to judge its weight from the action kinematics alone (similar to: Pobric and Hamilton, 2006) while under the effect of cTBS. cTBS (compared with SHAM) over BA1/2 impaired the accuracy with which participants judged the actions of others (N. Valchev, E. Tidoni, A. Hamilton, V. Gazzola, and A. Avenanti, in preparation; Primary Somatosensory cortex necessary for the perception of weight from other people’s action: a continuous theta-burst cTBS experiment). Interestingly, no impairments were detected after stimulation of nearby control sites 2.5-cm anterior or posterior to BA1/2. Hence, perturbation effects of cTBS on action perception may be site-specific, although not site-limited as suggested by the present study. Third, in our experiment we use the same fMRI data to assess the effect of cTBS over SI and regress the distal effects on the pMNS. It is therefore difficult to disentangle the contribution of cTBS from that of random fluctuations in brain responses across days. However, the significant difference in standard deviations between contrasts with and without cTBS confirms that cTBS has contributed to the effects we see. Collecting fMRI independent data to assess the effect of cTBS in a particular participant (e.g. somatosensory-evoked-potentials) might help better disentangling the specific effect of cTBS. Fourth, our regression approach can reveal relationship between cTBS-induced changes in BA1/2 and changes induced in connected brain regions if these are linear (be they inhibitory or excitatory), but may have missed more complex non-linear relationships.

Recently, it has been argued that action observation may involve mechanisms to predict future visual input using feedback information from the pMNS to the high-level visual areas of the MTG (Keysers *et al.*, 2004; Gazzola and Keysers, 2009; Kilner *et al.*, 2009; Schippers *et al.*, 2010; Friston *et al.*, 2011; Avenanti *et al.*, 2013a; Keysers and Gazzola, 2014). The fact that we found regions of the MTG to be influenced after cTBS on BA1/2 speaks in favor of the presence of such causal feedback influences onto the MTG.

In conclusion, by harnessing the capacity of cTBS to alter brain activity in remote interconnected regions, we provide evidence that the pMNS exchanges action-observation-specific information with BA1/2. This suggests closely integrating somatosensory and motor components in models of action observation. Rather than generating separate somatosensory and motor representations of the actions of others, the brain seems to take advantage of the tight connections between the somatosensory and motor cortex, evolved for motor control, to generate integrated sensorimotor vicarious representations. In addition, our study refines our understanding of the utility of offline cTBS in fMRI connectivity analyses, by showing that this type of cTBS increases between-subject variance (but not within; Supplementary Section ‘Within subjects variance, and global differences’), and thereby the efficiency with which a network of distal influences can be detected across days.

## Supplementary Material

Supplementary Data
