# Supplementary material for: Primary somatosensory contribution to action observation brain activity—combining fMRI and cTBS
Source: Soc Cogn Affect Neurosci. 2016 Mar 15;11(8):1205–17. doi: 10.1093/scan/nsw029 (PMC4967793; doi:10.1093/scan/nsw029)
Supplement: Supplementary Data [file supp_11_8_1205__index.html]

Primary somatosensory contribution to action observation brain activity—combining fMRI and cTBS — Primary somatosensory contribution to action observation brain activity—combining fMRI and cTBS — Supplementary Data 

# Primary somatosensory contribution to action observation brain activity—combining fMRI and cTBS

## Supplementary Data

files

- Supplementary Data - docx file
