# Supplementary material for: Primary somatosensory contribution to action observation brain activity—combining fMRI and cTBS
Source: Soc Cogn Affect Neurosci. 2016 Mar 15;11(8):1205–17. doi: 10.1093/scan/nsw029 (PMC4967793; doi:10.1093/scan/nsw029)
Supplement: Supplementary Data [file supp_nsw029_scan-15-412-File005.docx]

**Supplemental Figures**

**
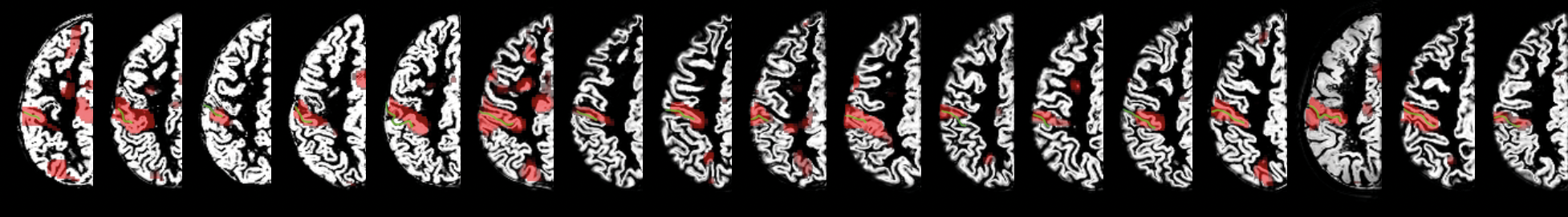
**

**Figure S1. Evidence for BA2 activation by the sight of actions.** For each of the 17 participants, the figure shows the left hemisphere gray matter segment of that participant after normalization to MNI in white, together with results of the contrast ActionObs-CtrlObs thresholded at p<0.001 uncorrected from the first level analysis of that participant in red. All slices are shown at z=44, which is the peak activation in SI in the group analysis. In green, an approximation of the postcentral sulcus to guide the reader. Activation during the sight of actions includes the anterior bank of the postcentral sulcus known to contain BA2.

**
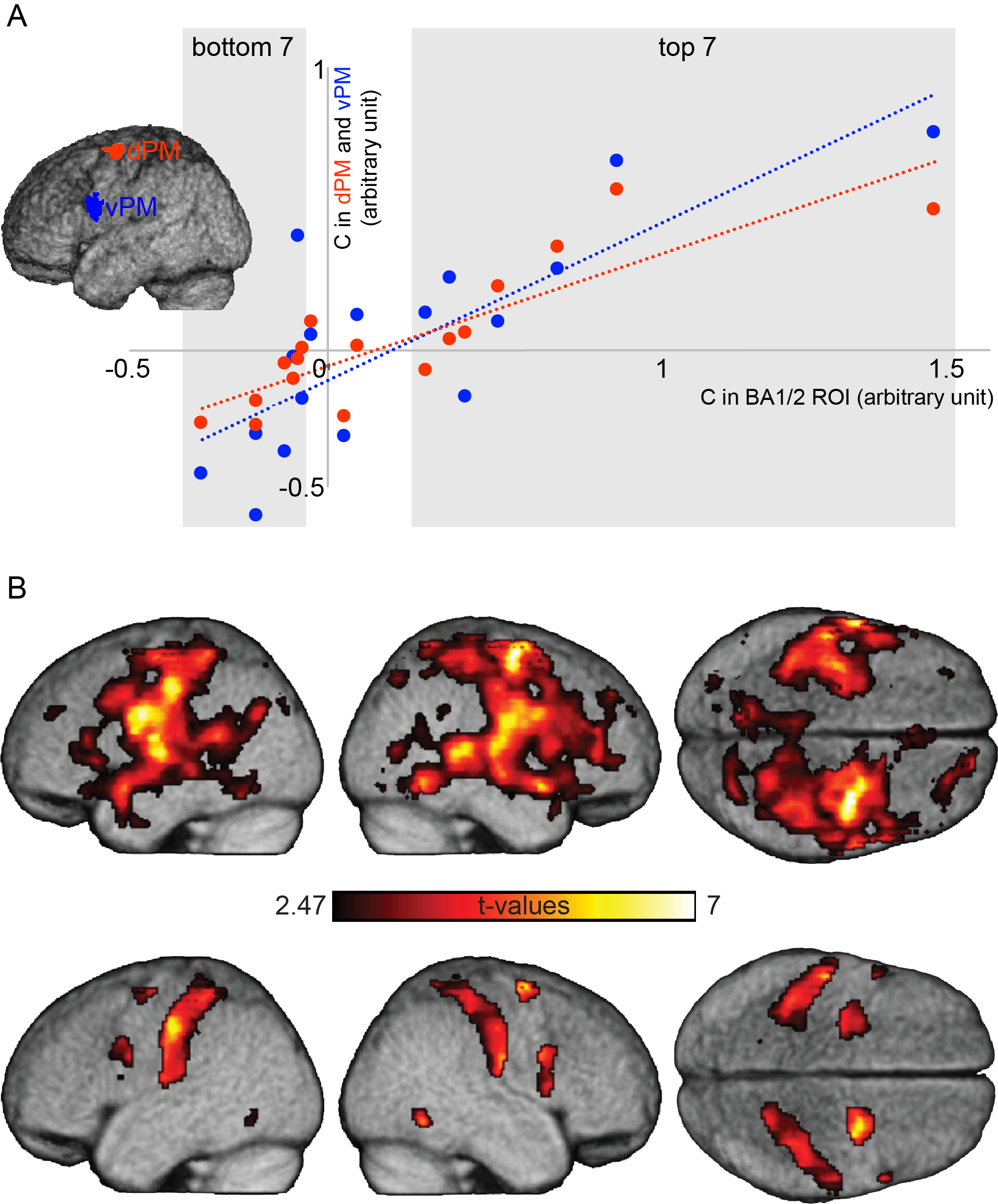
Figure S2. Remote cTBS effect – Group differences.**

**A**. For each of the 17 participant we extracted the value of C in the BA1/2 ROI under the coil (x-axis), and in the dPM and vPM ROIs (y-axis, as defined in the methods section and shown in the render). The trendline illustrates the linear relationship we exploit in the main paper. As an alternative analysis, we also split participants based on whether cTBS lead to an increase (C>0) or a decrease (C<0) in action observation specific activation in BA1/2. Seven participants showed substantial increases in activation (i.e. C>>0), and were classified as Top 7 responders in terms of activation under the coil (i.e. in the BA1/2 ROI). For numerical symmetry, we then also identified the Bottom 7 participants, all showing C<0. The remaining 3 participants have values close to zero, and were left out of this analysis. We then performed a t-test comparing the C values in dPM and vPM across these two groups. This revealed significant differences in dPM and vPM values (both p<0.01). Performing that analysis with the top 8 and bottom 8 in terms of BA1/2 activity also leads to significant differences in dPM and vPM activity. This shows that participants that responded to cTBS with an increase of activity in BA1/2 showed a significant increase in activity in dPM and vPM relative to those responding to cTBS with a decrease of activity in BA1/2.

**B.** Whole brain group comparison between Top7 and Bottom7. Classifying participants as in (A) based on the BA1/2 C values in these two groups of seven, and comparing their voxel-wise C values across the entire brain reveals widespread significant differences (top row). As can be seen in the bottom row, masking these results inclusively with the shared circuit mask (taken from Figure 2A) confirms that the dorsal and ventral premotor cortices bilaterally respond as would be expected if they received input from BA1/2. All results shown at q_fdr_<0.05. Computing the opposite contrast, looking for brain regions that showed activity *increases* in those participants that showed activity *decreases* in BA1/2 revealed no significant voxels at q<0.05.


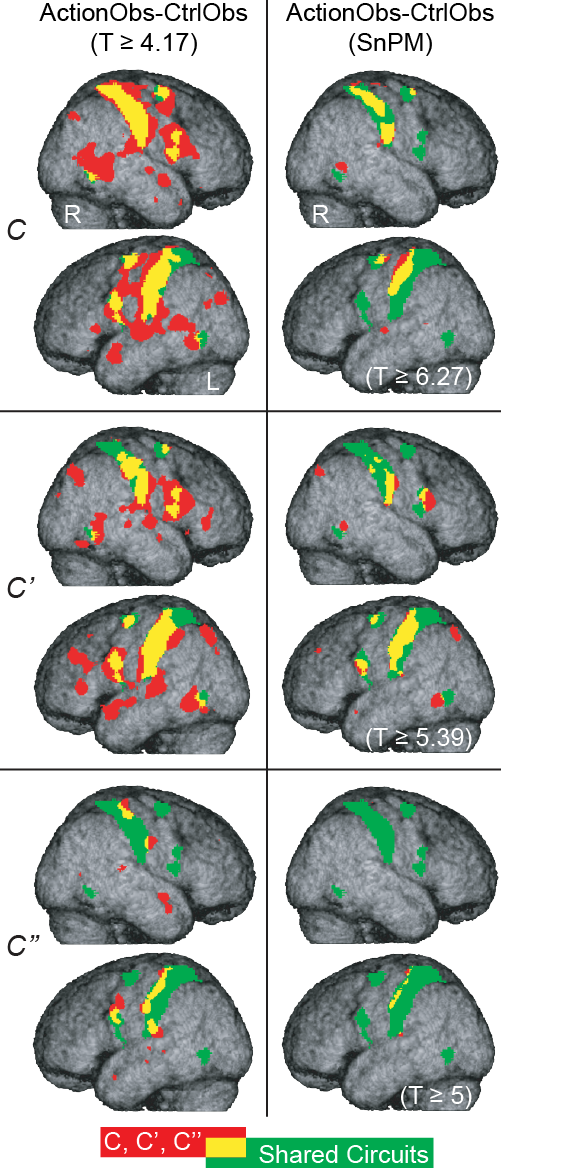
**Figure S3**. **Additional regression analyses results for C, C' and C''.** The first column on the left: same as in Figure 2B-D, but all at T≥4.17 in all cases (see also Supplemental section ‘*Statistical maps thresholding considerations*’). Second column: non-parametric statistic results. In the light of changes in the distribution of C due to cTBS, we replicated the analyses shown in Figure 2B-D using non-parametric tests (SnPM). As expected, given that non-parametric analyses have less statistical power, SnPM revealed more restricted networks, but confirmed that regressions involving cTBS (C and C’) predict premotor activations changes in the hemisphere ipsilateral to the cTBS perturbation, while those not involving cTBS (C’’), did not. For each contrast (C, C’ and C’’) the pseudo T-maps for the negative and positive effects were computed at p_FWE_ <0.05 (5000 permutations). As for the parametric analysis, the shared-voxels-activations-changes predicted by the BA1/2-ROI-activation-changes using C and C’ significantly outnumbered those predicted using C’’ (Chi-Square test, both p_(one tailed)_<0.0001).

**
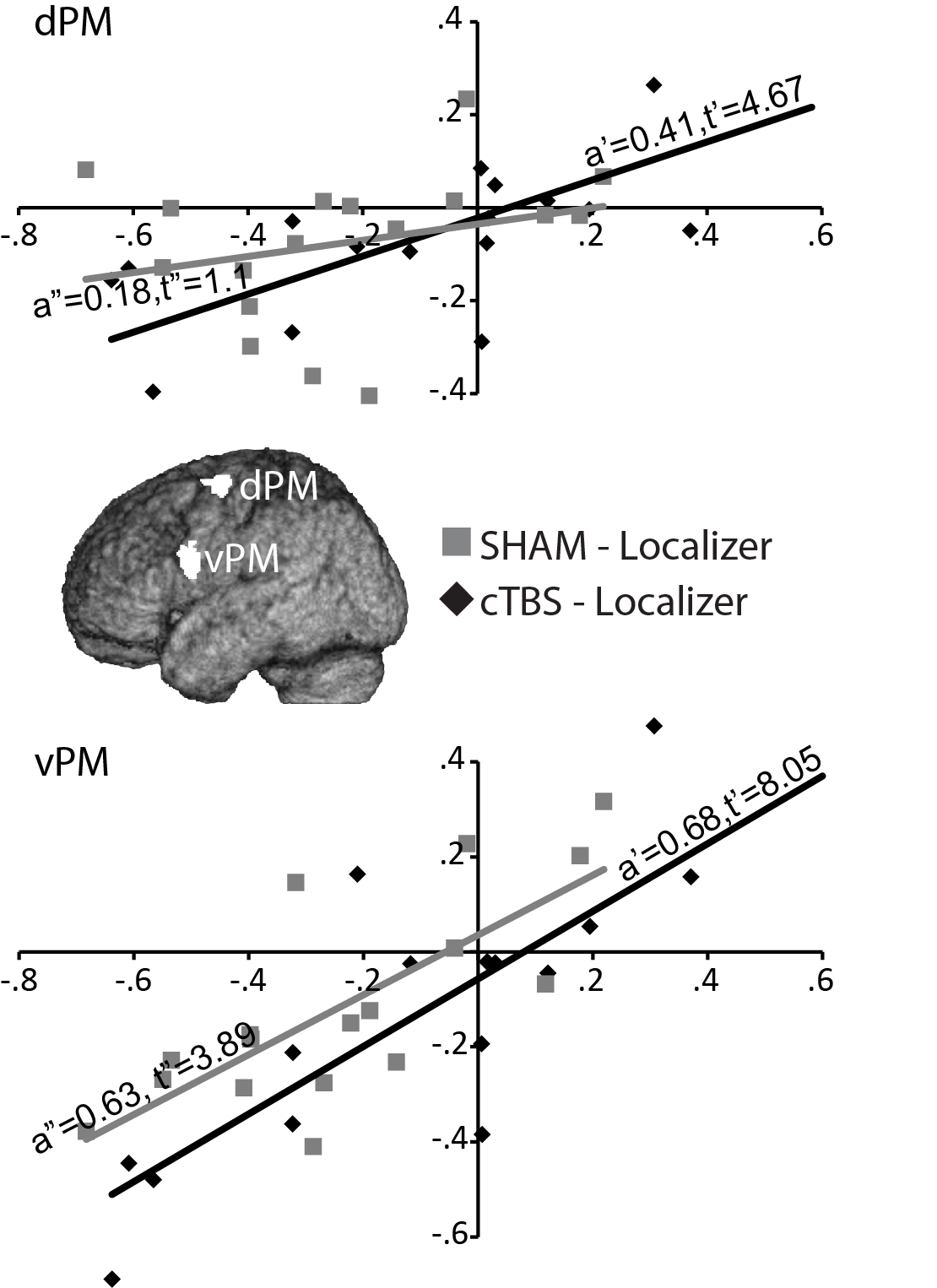
Figure S4.** **Graphic illustration of the distal effect of cTBS on ventral (vPM) and dorsal (dPM) premotor cortices.** Black lines: mean C’ signal in left (ipsilateral to the cTBS) dorsal and ventral premotor (dPM, vPM) nodes of the shared circuit network plotted against C’_BA1/2_. Grey lines: same but for C’’. The actual slopes of the black and grey regression lines look similar, and comparing them using a multiple regression did not yield significant differences for the dPM (F≥2.02, p≤0.17) or vPM (F≥0.24, p≤0.63) ROI. However, the larger spread of values along the x-axis in the case involving cTBS (black) probes the relation between BA1/2 and dPM/vPM more effectively, as revealed by the larger t values (i.e. t’>t”). All simple regressions were repeated using robust regressions (Robustfit in matlab; yielding the t’ and t”, and a’ and a’’ reported in the graph, for regressions based on C’ and C”, respectively) to avoid that the subject to the very left influences the regression unduly. This robust regression confirms the significant stimulus dependent connectivity based on C’, with p<0.001 for both dPM and vPM. The same connectivity when using the regression based on C”, with its smaller standard deviation, is no longer significant at α=0.001, with p>0.28 (dPM) and p<0.002 (vPM). Doing the same robust regression for C revealed significant connectivity between SI and both dPM and vPM (both p<0.0002, Figure S2a).

**Supplemental Table**

**Table S1.** **Experimental video-stimuli.** List of the hand actions used in the ActionObs. The actor’s right hand always entered the screen and initiated the goal-directed action from the left side of the screen. Note that CtrlObs movies showed the same objects used for ActionObs but the actor’s hand moved close to but without interacting with the object.

| **N◦** | **Recorded actions description** | **Movie**  **Duration** |
| --- | --- | --- |
| 1 | Stirring coffee with a spoon. | 3s |
| 2 | Putting a cube of sugar in a cup of coffee. | 3s |
| 3 | Closing a box with a key. | 3s |
| 4 | Lighting a candle with a lighter. | 3s |
| 5 | Putting a flower in a vase | 3s |
| 6 | Putting a battery in a remote control. | 3s |
| 7 | Putting a CD in a CDs stack. | 4s |
| 8 | Hammering a nail. | 4s |
| 9 | Putting whipped cream on strawberries. | 4s |
| 10 | Cutting a deck of cards. | 3s |
| 11 | Placing jewelry in a box. | 3s |
| 12 | Crumpling a paper sheet. | 3s |
| 13 | Closing a box of chewing gums. | 3s |
| 14 | Putting a pin on a foam base. | 3s |
| 15 | Taking hand cream from a tin. | 3s |
| 16 | Taking some tape and placing it on a box. | 4s |
| 17 | Pouring wine in a glass | 4s |
| 18 | Watering a plant. | 4s |
| 19 | Stirring eggs. | 3s |
| 20 | Closing a water bottle. | 3s |
| 21 | Flipping through a block note. | 3s |
| 22 | Taking an olive from a jar. | 3s |
| 23 | Putting a candle in a candleholder. | 3s |
| 24 | Closing a folder. | 3s |
| 25 | Cracking walnuts. | 4s |
| 26 | Placing a wine bottle in a box. | 4s |
| 27 | Opening a suitcase. | 4s |
| 28 | Spreading jam on a piece of bread. | 3s |
| 29 | Cutting a ribbon on a package. | 3s |
| 30 | Stirring soup with a spoon. | 3s |
| 31 | Putting business cards in a box. | 3s |
| 32 | Putting a hair clip in a purse. | 3s |
| 33 | Disconnecting headphones from an MP3 player. | 3s |
| 34 | Breaking an egg on the edge of a bowl. | 4s |
| 35 | Stirring a painting brush in a cup of water. | 4s |
| 36 | Taking a walnut with chopsticks and placing it in a box. | 4s |

**Supplemental methods and analyses**

***Statistical maps thresholding considerations.***

We then used the SPM fdr correction to determine the t-threshold with a false-positive rate of 5% for each contrast (t_q=0.05_). If t_q=0.05_< t_p=0.001_ (which was true in most cases), results are still presented at t_p=0.001,_specifying that results survive fdr correction. In these cases t_p=0.001_ was used to permit comparison across contrasts, rather than t_q=0.05_, which varies from contrast to contrast, to permit a fair comparison of results across contrasts. False positive rates remain controlled to remain below 0.05. If t_q=0.05_> t_p=0.001_ (rarely the case), the situation becomes more difficult. In the interest of controlling false positive rates to remain below 5%, it is then unacceptable to use t_p=0.001_, but if one then uses t_q=0.05_ that varies from contrast to contrast, it becomes difficult to compare effect sizes across thresholded maps. Hence, we show maps at t_q=0.05_ in the main text (to ensure false positive rate < 5%), and show the other contrasts that need to be compared with that contrast the same t-threshold in the supplementary materials to permit comparison of effect sizes (in full awareness of the fact that these specific t-values are not t_q=0.05_ for those other contrasts. These threshold dependent analysis are complemented with threshold independent analysis for further validation.

***Changes in connectivity (PPI analysis)***.

The notion that the connectivity between BA1/2 and the rest of the brain was changed by cTBS was further tested by performing a PPI analysis. For each subject, we created a first level model including the SHAM and cTBS sessions, from which we extracted the first eigen-value of the BA1/2 single subject's ROIs. Two separate psycho-physiological interactions (PPI; Friston et al. 1997) were then performed. In one case, a weight of 1 was given to the blocks of cTBS-ActionObs and a weight of -1 to SHAM-ActionObs, to examine how connectivity from BA1/2 changed during action observation as a function of cTBS. In the other case, a weight of 1 was given to cTBS-CtrlObs and -1 to SHAM-CtrlObs to examine connectivity changes during control observation. The interaction parameter estimates for each PPI were then taken to the second level. One-sample t-tests comparing the interaction parameter estimates against zero for ActionObs or for CtrlObs did not reveal significant changes in connectivity anywhere in the grey matter (even at p_unc_<0.005, T(16)>2.9, minimum cluster-size 10). A two-sample t-test was also used to compare the interaction parameters of ActionObs and CtrlObs to examining whether changes in connectivity might have been different across the two conditions, and no significant differences were found in the gray matter (even at p_unc_<0.005, T(16)>2.9, min cluster size 10 voxels). Note that this two-sample t-test is equivalent to subtracting the PPI term for ActionObs and ControlObs, and testing the difference against zero, and is thus equivalent to calculating a PPI that contrasts ActionObs-CtrlObs, and then compare the PPI term between cTBS and Sham. The fact that no voxels showed significant alterations of functional connectivity with BA1/2 between the cTBS and SHAM condition suggests that cTBS induced changes in functional connectivity are modest compared to the gain in efficiency documented in the main text.

## *Within subjects variance, and global differences*

In order to establish if cTBS affected the goodness of fit of the GLM we compared the residual errors from first-level GLMs fitted to the SHAM and cTBS data using non-parametric permutation tests across subjects (SnPM, because the sums or squares of errors are not normally distributed), and found no significant differences (q_fdr_>0.05). This suggests that cTBS increased the variance across subjects (at the second level), but not the variance in the responses across trials (at the first level).

We then explored if the effect of cTBS changed over the time of our fMRI session. We modeled each ActionObs and CtrlObs block separately to generate a single parameter estimate for each occurrence of ActionObs and for each occurrence of CtrlObs. We then calculated C in each voxel *j* separately for each occurrence (i.e. contrasting the first occurrence of ActionObs with CtrlObs, then doing the same for the second occurrence etc.), and used an ANOVA with 36 occurrences to see if C changed as a function of occurrence. We did not find evidence for such an effect at q_fdr_<0.05.

The global parameter from the cTBS and SHAM session (i.e. the time-constant parameters in the GLM) were compared with a t-test to examine if cTBS systematically altered baseline activity. No significant differences were found between these sessions (whole brain, q_fdr_>0.05).
